# Characteristics of early‐onset pancreatic cancer and its association with familial pancreatic cancer and hereditary pancreatic cancer syndromes

**DOI:** 10.1002/ags3.12326

**Published:** 2020-03-27

**Authors:** Hidetoshi Eguchi, Shogo Kobayashi, Kunihito Gotoh, Takehiro Noda, Yuichiro Doki

**Affiliations:** ^1^ Department of Gastroenterological Surgery Graduate School of Medicine Osaka University Osaka Japan

**Keywords:** early‐onset pancreatic cancer, familial pancreatic cancer, hereditary, pancreatic cancer syndromes

## Abstract

The incidence of pancreatic cancer is high among those in their sixties to seventies but low in those in their fifties or younger. Although there is no unified definition regarding the age of early‐onset pancreatic cancer, previously published reports suggest that, compared to later‐onset pancreatic cancer patients, early‐onset pancreatic cancer patients tend to be detected at advanced stages and thus have poor prognoses, but they do not show significantly higher rates of patients with genetic factors. On the other hand, it has been reported that patients with familial pancreatic cancer and hereditary pancreatic cancer syndromes often develop pancreatic cancer at a young age. The broad definition of familial pancreatic cancer is pancreatic cancer in patients who have two or more first‐degree relatives with pancreatic cancer; whereas the narrow definition of familial pancreatic cancer is the broad definition of familial pancreatic cancer, while excluding those with inherited tumor syndromes. Hereditary tumors developing pancreatic cancer include hereditary pancreatitis, hereditary breast and ovarian cancer, Peutz‐Jeghers syndrome, familial atypical multiple mole melanoma syndrome, familial adenomatous polyposis, and hereditary non‐polyposis colorectal cancer, all of which are autosomal dominant hereditary diseases. This study reviews the clinical characteristics of early‐onset pancreatic cancer and its association with familial pancreatic cancer and hereditary pancreatic cancer syndromes.

## Graphical abstract

The incidence of pancreatic cancer is high among those in their sixties to seventies but low in those in their fifties or younger. This study provides an overview of the clinical characteristics of early‐onset pancreatic cancer and its association with familial pancreatic cancer and hereditary pancreatic cancer syndromes.

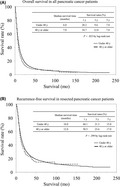

## 1 INTRODUCTION

Despite an improved survival rate among cancer patients due to recent advancements in medical science, the prognosis of pancreatic cancer is still very poor. In the US and Japan, the number of deaths from pancreatic cancer is the fourth largest among all types of cancer, and is an intractable cancer with a 5‐year survival rate of less than 10%.^1^ Pancreatic cancer is difficult to diagnose in the early stages. In many patients, symptoms are not exhibited until the cancer has already become advanced. A medical check‐up, even without symptoms, for the detection of pancreatic cancer at an early stage was therefore carried out, with the aim of improving the survival rate of patients with this disease. Clarification of the risk factors for pancreatic cancer and careful examination for those with these risk factors is vital for effective examinations.

Many studies have focused on familial pancreatic cancer and hereditary pancreatic cancer syndromes. And, of course, aging is a significant risk factor. The incidence of pancreatic cancer is high among those in their sixties to seventies but low in those patients in their fifties or younger.^2^ Is early‐onset pancreatic cancer just an accidental disease? Is there any reason for early onset? These questions may be crucial in order to clarify the pathogenesis of pancreatic cancer. However, there have been few studies on early‐onset pancreatic cancer. The etiology and pathology of early‐onset pancreatic cancer is important information for the determination and explanation of therapeutic strategies among patients thereof. This study provides an overview of the clinical characteristics of early‐onset pancreatic cancer along with familial pancreatic cancer and hereditary pancreatic cancer syndromes.

## 2 EARLY‐ONSET PANCREATIC CANCER

There is no unified definition regarding the age of early‐onset pancreatic cancer. Various definitions have been used in studies conducted by different researchers (under the age of 40, 45, 50, or 60). Raimondi et al^3^ defined the age of early‐onset pancreatic cancer as under 50 and demonstrated that the incidence of early‐onset pancreatic cancer is high in countries with high smoking rates based on a comprehensive analysis of WHO data, SEER data, and references from PubMed. They reported that smoking plays a significant role in the incidence of early‐onset pancreatic cancer, based on a positive correlation between the incidence of early‐onset pancreatic cancer and the mortality of lung cancer, along with the fact that males with a higher smoking rate have a high incidence of early‐onset pancreatic cancer. Upon further conducting a literary review, they concluded that genetic factors and smoking are involved in early‐onset pancreatic cancer (EOPC).

Piciucchi et al^4^ defined the onset of pancreatic cancer at an age of 50 or younger as EOPC and compared the characteristics of 25 patients suffering from EOPC with 268 patients with later‐onset pancreatic cancer (LOPC). They reported that being a smoker and starting smoking at a young age are independent risk factors of EOPC and that there was no significant difference in the incidence of familial pancreatic cancer and hereditary pancreatic cancer syndromes. According to this report, although EOPC tends to be discovered as an unresectable advanced cancer, aggressive surgery and chemotherapy may result in similar prognoses to that of LOPC.

**Figure 1:**
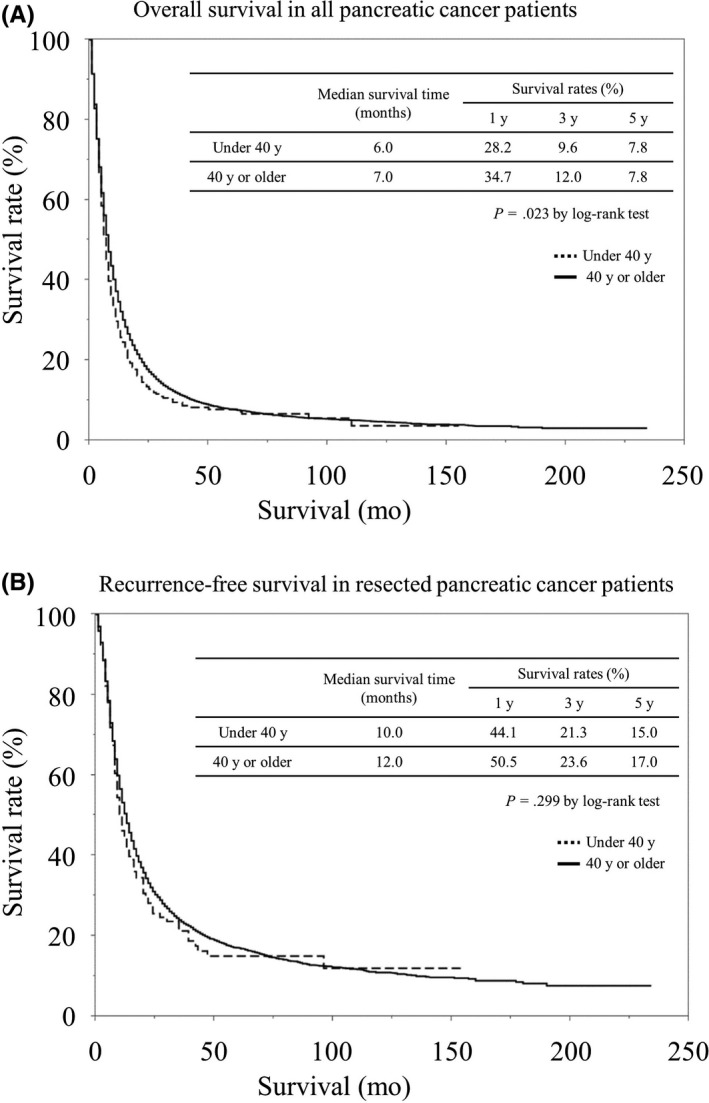
A, Comparison of overall survival rates between EOPC and LOPC in the Pancreatic Cancer Registry of the Japan Pancreas Society.^5^ B, Comparison of Recurrence‐free survival rates.^5^

It is easier to recruit patients with EOPC to a study when patients with EOPC are defined as those younger than 50 or 60 years of age, but these definitions may fail to identify the characteristics that are specific to young patients with PDAC. Thus, we used data from The Pancreatic Cancer Registry of the Japan Pancreas Society to compare patients suffering from EOPC (onset at an age under 40) with those who developed pancreatic cancer at 40 or older and examined the clinicopathological differences.^5^ Among the 36 145 cases registered in The Pancreatic Cancer Registry, 526 patients developed pancreatic cancer under the age of 40 (1.5%). There was no significant difference between these patients and those who developed pancreatic cancer over the age of 40 in terms of their family history of pancreatic cancer or their smoking or drinking history. Patients who developed pancreatic cancer under the age of 40 tended to have larger tumor sizes, liver metastases, peritoneal dissemination, and distant lymph node metastases, with a significantly lower rate of pancreatectomy. Due to these background factors, the overall survival among pancreatic cancer patients under the age of 40 was significantly worse. However, a comparison between the two groups of patients who underwent resection revealed no significant difference in overall survival (Figure 1).

On the other hand, Ansari et al^6^ reviewed 72 906 cases pathologically diagnosed with pancreatic ductal carcinoma from the data of The Surveillance, Epidemiology, and End Results (SEER) between 2004 to 2016 to compare the EOPC group with onset at the age of 50 or younger and the LOPC group with onset at the age of 50 or older. The EOPC group included 4523 cases (6.2%). The detection of EOPC tended to be at the advanced AJCC stages and the frequency of surgery, radiotherapy, and chemotherapy was higher in EOPC patients. Comparing the EOPC and LOPC groups using propensity score matching, the EOPC group had a significantly worse overall survival rate (OS) after 5 years (6.1% vs 8.6%, *P* = .003) as well as a significantly worse cancer‐specific survival rate (CSS) (6.7% vs 9.7%, *P* < .001). A comparison only among those who underwent surgery indicated worse OS and CSS in the EOPC group as well. Based on the above results, the authors concluded that EOPC has different tumor biological characteristics from LOPC. This fact may be important information in the determination and explanation of therapeutic strategies for the EOPC group.

These reports suggest that, compared to LOPC patients, EOPC patients tend to be detected at advanced stages and have worse prognoses, but do not show significantly higher rates of patients with genetic factors.

## 3 FAMILIAL PANCREATIC CANCER

While some studies have reported that EOPC patients do not have a significantly higher rate of patients with genetic factors such as familial pancreatic cancer or hereditary pancreatic cancer syndromes, others have reported that patients with familial pancreatic cancer and hereditary pancreatic cancer syndromes often develop pancreatic cancer at a young age.^7^, ^8^ On the other hand, it has also been reported that there is no difference in the age of onset.^9^

Approximately 10% of pancreatic cancer patients reportedly have a family history.^10^, ^11^ In a Japanese study, 88 out of 1197 cases had first‐degree relatives with pancreatic cancer (7.3%), indicating a rate of familial pancreatic cancer among pancreatic cancer patients similar to the results of Western studies.^9^ The broad definition of familial pancreatic cancer is pancreatic cancer in patients who have two or more first‐degree relatives with pancreatic cancer.^12^ The narrow definition of familial pancreatic cancer is the broad definition of familial pancreatic cancer but excluding those with inherited tumor syndromes.^13^, ^14^ A study from the US reported that the standardized incidence ratio (SIR) of patients with one first‐degree relative was 4.5 fold higher, while that of patients with two first‐degree relatives was 6.4 fold higher, and that of patients with three first‐degree relatives was 32 fold higher, indicating a higher risk of pancreatic cancer.^12^

Genetic abnormalities are found in only 20% of patients with familial pancreatic cancer.^15^ Takai et al^9^ examined 21 genetic mutations involving hereditary pancreatic cancer syndromes among 54 cases of familial pancreatic cancer in which germ cell DNA could be collected. As a result, there were three cases of *BRCA2* mutation (5.6%), two cases of *PALB2* mutation (3.7%), two cases of *ATM* mutation (3.7%), and one case of *MLH1* mutation (1.9%). A US study reported on 17 cases of *BRCA1/2* mutation (11.3%) detected from genetic testing among 151 pancreatic cancer cases.^16^ According to a Canadian cohort study, 4.6% of pancreatic cancer patients had deleterious mutations in the *BRCA* genes and pancreatic cancer patients with deleterious mutations in *BRCA* genes exhibited no differences with patients without them in terms of age at diagnosis for pancreatic cancer, sex, or smoking history.^17^

Patients with familial pancreatic cancer account for 5%‐10% of all patients with pancreatic cancer.^15^ There is no significant difference between familial and sporadic pancreatic cancer in terms of onset location, symptoms, imaging findings, stage of pancreatic cancer, or pathological findings. Treatment for the two is basically the same. However, since it has been reported that platinum‐based chemotherapy and poly ADP‐ribose polymerase (PARP) inhibitors are effective for pancreatic cancer with *BRCA2* genetic mutations and *PALB2* genetic mutations (but not covered by insurance for pancreatic cancer), genetic testing may enable the selection of treatment based on precision medicine.^18^

Johns Hopkins Hospital carried out computed tomography (CT) and endoscopic ultrasound (EUS) screening tests among 78 subjects with a high risk of pancreatic cancer including 72 subjects in a familial pancreatic cancer family line and 161 control subjects, reporting eight subjects (10%) with a high risk of pancreatic cancer having neoplastic lesions, which was a high incidence.^19^ Such medical examination for pancreatic cancer among subjects in a familial pancreatic cancer family line may be effective. The International Cancer of the Pancreas Screening (CAPS) Consortium reported recommended methods for screening people at risk for familial pancreatic cancer.^20^ According to this recommendation, surveillance should start no earlier than age 50 or 10 years earlier than the youngest relative with pancreatic cancer, but were split on whether to start at age 50 or 55. Preferred surveillance tests are EUS and magnetic resonance imaging (MRI)/magnetic retrograde cholangiopancreatography. Annual surveillance is recommended in the absence of concerning lesions.

## 4 HEREDITARY PANCREATIC CANCER SYNDROMES

According to the narrow definition of familial pancreatic cancer, subjects with hereditary tumors are not included in familial pancreatic cancer. This section illustrates hereditary tumors developing pancreatic cancer. Hereditary tumors developing pancreatic cancer include hereditary pancreatitis, hereditary breast and ovarian cancer (HBOC), Peutz‐Jeghers syndrome (PJS), familial atypical multiple mole melanoma syndrome (FAMMM), familial adenomatous polyposis (FAP), and Lynch syndrome (hereditary non‐polyposis colorectal cancer [HNPCC]), each of which are autosomal dominant hereditary diseases (Table 1).^21^

**Table 1:** Hereditary pancreatic cancer syndromes

Disease	Gene	Risk ratio
Hereditary pancreatitis	*PRSS1, SPINK1, CFTR, CTRC*	60‐87
Hereditary breast and ovarian cancer; HBOC	*BRCA1/2*	4.1‐5.8
Peutz‐Jeghers syndrome; PJS	*STK11/LKB1*	132
Familial atypical multiple mole melanoma syndrome; FAMMM	*CDKN2A/p16*	13‐22
Familial adenomatous polyposis; FAP	*APC*	4.5
Hereditary non‐polyposis colorectal cancer; HNPCC (Lynch syndrome)	*MLH1, MSH2, MSH6, PMS2*	8.6

### 4.1 Hereditary pancreatitis

Hereditary pancreatitis was defined as inflammation of the pancreas, usually recurrent from childhood, with unusual prevalence among blood‐related groups of persons in accordance with Mendelian laws.^22^ As causative genes of hereditary pancreatitis, *PRSS1, CFTR, SPINK1, *and *CTRC* have been reported.^22^ The onset risk of pancreatic cancer in patients with this disease is 10.0% and 53.5% at the age of 50 and 75, respectively, which are 60 to 87 fold higher than that of ordinary people; in particular, the average age of the onset of pancreatic cancer is 20 years younger in those with a history of smoking, with smoking reportedly significantly increasing the risk therefor.^8^, ^23^ In relation to carcinogenesis, these gene mutations are thought to cause continuous chronic pancreatitis from childhood, leading to the formation of precancerous lesions.^24^

### 4.2 Hereditary breast and ovarian cancer

Hereditary breast and ovarian cancer is an autosomal dominant genetic disease characterized by a mutation in the *BRCA 1/2* genes. *BRCA* genes involve Fanconi anemia/BRCA pathways and are responsible for DNA repair. The onset risk of pancreatic cancer in patients with a *BRCA* gene mutation is 4.1 to 5.8‐fold higher.^25^ Therefore, patients diagnosed with HBOC and their families should undergo careful screening not only for breast cancer and ovarian cancer but also for pancreatic cancer.

### 4.3 Peutz‐Jeghers syndrome

This disease is characterized by hamartomatous gastrointestinal polyposis and mucosal pigmentation, involving pigmentation, ileus, abdominal pain, bloody stool, and intussusception due to small intestinal polyps. It is caused by the deficiency of *SKT11/LKB1* genes. This disease involves a high risk of gastrointestinal and extragastrointestinal malignant tumors and a 132‐fold higher risk of pancreatic cancer.^26^

### 4.4 Familial atypical multiple mole melanoma syndrome

Compared to non‐familial malignant melanoma, this disease has the following characteristics: relatively early‐onset; more frequent onset of primary malignant melanoma; relatively better prognosis; a higher incidence of other malignancies in the same family line; higher frequency of superficial spreading melanoma; and a difference in incidence depending on race. The *CDKN2A* gene mutation is one of the typical gene mutations triggering this disease. The onset risk of pancreatic cancer among FAMMM family lines is reportedly 13 to 22‐fold higher.^27^, ^28^

### 4.5 Familial adenomatous polyposis

This disease involves a high incidence of colorectal cancer due to multiple adenomas in the large intestine. It may be complicated with adenomas and cancers of the stomach, duodenum, and small intestine, as well as tumors in organs other than the gastrointestinal tract such as bone tumors, desmoid tumors, papillary thyroid carcinoma, medulloblastoma, and retinal pigment degeneration. The causative gene is *APC* gene. The onset risk of pancreatic cancer among patients in FAP family is reportedly 4.5‐fold higher.^29^

### 4.6 Lynch syndrome (hereditary non‐polyposis colorectal cancer)

The genes which code the proteins responsible for the repair of DNA replication error are called mismatch repair (MMR) genes. Reduced repair function due to abnormalities in these MMR genes causes cancer. Lynch syndrome is caused by the DNA mismatch repair genes (*MLH1*, *MSH2, MSH6, PMS2*) involving colorectal cancer, endometrial cancer, stomach cancer, ovarian cancer, pancreatic cancer, ureteral cancer, renal pelvis cancer, biliary tract cancer, glioblastoma, and small intestine cancer. The onset risk of pancreatic cancer among patients with this disease is reportedly 8.6‐fold higher.^30^

## 5 CONCLUSION

Effective screening and diagnosis is required at an early stage of pancreatic cancer, which still has a poor prognosis. To this end, it is important to elucidate the risk factors for pancreatic cancer. Early‐onset pancreatic cancer, familial pancreatic cancer, and hereditary pancreatic cancer syndromes are relatively rare pancreatic cancers. However, epidemiologic, pathological, and genetic studies of these patients may reveal other risk factors for pancreatic cancer.

## DISCLOSURE

Conflict of interest: The authors declare no conflict of interest for this article.

## References

[1] Siegel RL , Miller KD , Jemal A . Cancer statistics, 2019. CA Cancer J Clin. 2019;69(1):7–34. 30620402 10.3322/caac.21551

[2] Raimondi S , Maisonneuve P , Lowenfels AB . Epidemiology of pancreatic cancer: an overview. Nat Rev Gastroenterol Hepatol. 2009;6(12):699–708. 19806144 10.1038/nrgastro.2009.177

[3] Raimondi S , Maisonneuve P , Lohr JM , Lowenfels AB . Early onset pancreatic cancer: evidence of a major role for smoking and genetic factors. Cancer Epidemiol Biomarkers Prev. 2007;16(9):1894–7. 17855711 10.1158/1055-9965.EPI-07-0341

[4] Piciucchi M , Capurso G , Valente R , Larghi A , Archibugi L , Signoretti M , et al. Early onset pancreatic cancer: risk factors, presentation and outcome. Pancreatology. 2015;15(2):151–5. 25708929 10.1016/j.pan.2015.01.013

[5] Eguchi H , Yamaue H , Unno M , Mizuma M , Hamada S , Igarashi H , et al. Clinicopathological characteristics of young patients with pancreatic cancer: an analysis of data from Pancreatic Cancer Registry of Japan Pancreas Society. Pancreas. 2016;45(10):1411–7. 27171511 10.1097/MPA.0000000000000636

[6] Ansari D , Althini C , Ohlsson H , Andersson R . Early‐onset pancreatic cancer: a population‐based study using the SEER registry. Langenbecks Arch Surg. 2019;404(5):565–71. 31377855 10.1007/s00423-019-01810-0 PMC6713682

[7] James TA , Sheldon DG , Rajput A , et al. Risk factors associated with earlier age of onset in familial pancreatic carcinoma. Cancer. 2004;101(12):2722–6. 15534880 10.1002/cncr.20700

[8] Brune KA , Lau B , Palmisano E , Canto M , Goggins MG , Hruban RH , et al. Importance of age of onset in pancreatic cancer kindreds. J Natl Cancer Inst. 2010;102(2):119–26. 20068195 10.1093/jnci/djp466 PMC2808346

[9] Takai E , Yachida S , Shimizu K , Furuse J , Kubo E , Ohmoto A , et al. Germline mutations in Japanese familial pancreatic cancer patients. Oncotarget. 2016;7(45):74227–35. 27732944 10.18632/oncotarget.12490 PMC5342048

[10] Hruban RH , Canto MI , Goggins M , Schulick R , Klein AP . Update on familial pancreatic cancer. Adv Surg. 2010;44:293–311. 20919528 10.1016/j.yasu.2010.05.011 PMC2966038

[11] Bartsch DK , Gress TM , Langer P . Familial pancreatic cancer‐current knowledge. Nat Rev Gastroenterol Hepatol. 2012;9(8):445–53. 22664588 10.1038/nrgastro.2012.111

[12] Klein AP , Brune KA , Petersen GM , Goggins M , Tersmette AC , Offerhaus GJA , et al. Prospective risk of pancreatic cancer in familial pancreatic cancer kindreds. Cancer Res. 2004;64(7):2634–8. 15059921 10.1158/0008-5472.can-03-3823

[13] Benzel J , Fendrich V . Familial pancreatic cancer. Oncol Res Treat. 2018;41(10):611–8. 30269130 10.1159/000493473

[14] Fendrich V , Langer P , Bartsch DK . Familial pancreatic cancer‐status quo. Int J Colorectal Dis. 2014;29(2):139–45. 23948969 10.1007/s00384-013-1760-3

[15] Matsubayashi H , Takaori K , Morizane C , Maguchi H , Mizuma M , Takahashi H , et al. Familial pancreatic cancer: concept, management and issues. World J Gastroenterol. 2017;23(6):935–48. 28246467 10.3748/wjg.v23.i6.935 PMC5311103

[16] Salo‐Mullen EE , O'Reilly EM , Kelsen DP , Ashraf AM , Lowery MA , Yu KH , et al. Identification of germline genetic mutations in patients with pancreatic cancer. Cancer. 2015;121(24):4382–8. 26440929 10.1002/cncr.29664 PMC5193099

[17] Holter S , Borgida A , Dodd A , Grant R , Semotiuk K , Hedley D , et al. Germline BRCA mutations in a large clinic‐based cohort of patients with pancreatic adenocarcinoma. J Clin Oncol. 2015;33(28):3124–9. 25940717 10.1200/JCO.2014.59.7401

[18] Waddell N , Pajic M , Patch AM , Chang DK , Kassahn KS , Bailey P , et al. Whole genomes redefine the mutational landscape of pancreatic cancer. Nature. 2015;518(7540):495–501. 25719666 10.1038/nature14169 PMC4523082

[19] Canto MI , Goggins M , Hruban RH , Petersen GM , Giardiello FM , Yeo C , et al. Screening for early pancreatic neoplasia in high‐risk individuals: a prospective controlled study. Clin Gastroenterol Hepatol. 2006;4(6):766–81. 16682259 10.1016/j.cgh.2006.02.005

[20] Goggins M , Overbeek KA , Brand R , Syngal S , Del Chiaro M , Bartsch DK , et al. Management of patients with increased risk for familial pancreatic cancer: updated recommendations from the International Cancer of the Pancreas Screening (CAPS) Consortium. Gut. 2020;69(1):7–17. 31672839 10.1136/gutjnl-2019-319352 PMC7295005

[21] Okusaka T , Nakamura M , Yoshida M , et al. Clinical practice guidelines for pancreatic cancer 2019 from the Japan pancreas society: a synopsis. Pancreas. (In press). 10.1097/MPA.0000000000001513 PMC7077959 32132516

[22] Raphael KL , Willingham FF . Hereditary pancreatitis: current perspectives. Clin Exp Gastroenterol. 2016;9:197–207. 27555793 10.2147/CEG.S84358 PMC4968666

[23] Rebours V , Boutron‐Ruault MC , Schnee M , Férec C , Maire F , Hammel P , et al. Risk of pancreatic adenocarcinoma in patients with hereditary pancreatitis: a national exhaustive series. Am J Gastroenterol. 2008;103(1):111–9. 18184119 10.1111/j.1572-0241.2007.01597.x

[24] Weiss FU . Pancreatic cancer risk in hereditary pancreatitis. Front Physiol. 2014;5:70. 24600409 10.3389/fphys.2014.00070 PMC3929831

[25] Mocci E , Milne RL , Mendez‐Villamil EY , Hopper Jl , John EM , Andrulis Il , et al. Risk of pancreatic cancer in breast cancer families from the breast cancer family registry. Cancer Epidemiol Biomarkers Prev. 2013;22(5):803–11. 23456555 10.1158/1055-9965.EPI-12-0195 PMC3739843

[26] Giardiello FM , Brensinger JD , Tersmette AC , Goodman SN , Petersen GM , Booker SV , et al. Very high risk of cancer in familial Peutz‐Jeghers syndrome. Gastroenterology. 2000;119(6):1447–53. 11113065 10.1053/gast.2000.20228

[27] Rustgi AK . Familial pancreatic cancer: genetic advances. Genes Dev. 2014;28(1):1–7. 24395243 10.1101/gad.228452.113 PMC3894408

[28] Lynch HT , Fusaro RM , Lynch JF , Brand R . Pancreatic cancer and the FAMMM syndrome. Fam Cancer. 2008;7(1):103–12. 17992582 10.1007/s10689-007-9166-4

[29] Giardiello FM , Offerhaus GJ , Lee DH , Krush AJ , Tersmette AC , Booker SV , et al. Increased risk of thyroid and pancreatic carcinoma in familial adenomatous polyposis. Gut. 1993;34(10):1394–6. 8244108 10.1136/gut.34.10.1394 PMC1374548

[30] Kastrinos F , Mukherjee B , Tayob N , Wang F , Sparr J , Raymond VM , et al. Risk of pancreatic cancer in families with Lynch syndrome. JAMA. 2009;302(16):1790–5. 19861671 10.1001/jama.2009.1529 PMC4091624

